# Real World Outcomes of Ipilimumab and Nivolumab in Patients with Metastatic Melanoma

**DOI:** 10.3390/cancers12082329

**Published:** 2020-08-18

**Authors:** Nethanel Asher, Guy Ben-Betzalel, Shaked Lev-Ari, Ronnie Shapira-Frommer, Yael Steinberg-Silman, Neta Gochman, Jacob Schachter, Tomer Meirson, Gal Markel

**Affiliations:** 1 Ella Lemelbaum Institute for Immuno-Oncology, Sheba Medical Center, Ramat Gan 52621, Israel; Guy.Ben-Betzalel@sheba.health.gov.il (G.B.-B.); Shaked.LevAri@sheba.health.gov.il (S.L.-A.); Ronnie.Shapira@sheba.health.gov.il (R.S.-F.); Yael.Steinberg@sheba.health.gov.il (Y.S.-S.); netagochman@gmail.com (N.G.); jacob.schachter@sheba.health.gov.il (J.S.); tomermrsn@gmail.com (T.M.); 2 Sackler Faculty of Medicine, Tel Aviv University, Tel Aviv 6997801, Israel; 3 Azrieli Faculty of Medicine, Bar-Ilan University, Safed 1589, Israel; 4 The Department of Clinical Microbiology and Immunology, Sackler Faculty of Medicine, Tel Aviv University, Tel Aviv 6997801, Israel

**Keywords:** immunotherapy, programmed cell death 1 receptor, melanoma, CTLA-4 antigen, drug therapy-combination

## Abstract

*Background:* Immunotherapy has drastically changed the outlook for melanoma patients over the past decade. Specifically, the dual blockade of immune checkpoints using ipilimumab and nivolumab has shown unprecedented response rates and survival outcomes. This immense achievement, though, is at the cost of toxicity, with 60% of the patients experiencing high-grade adverse events (AEs). Our study aims to report the efficacy and toxicity outcomes of an out-of-trial, real-life population. *Methods:* Data on metastatic melanoma patients treated with ipilimumab and nivolumab were retrieved from our melanoma database—a single-center prospectively updated, medical-records based oncologic registry. Data included demographics, clinical and pathological information, as well as tumor responses and survival. Associations between patient or treatment characteristics and outcomes were also evaluated. *Results:* We identified 172 metastatic melanoma patients, of whom 64% were treatment-naïve. The median follow-up was 12 months. The response rates for treatment-naïve and previously-treated patients were 61% and 25%, respectively; median progression-free survival (PFS) were 12.2 and 2.6 months, and median overall survival (OS) were not-reached (NR) and 6.1 months, respectively. The estimated three-year OS for treatment-naïve patients was 58% (95% CI 42–65). At data cutoff, 22% were still on-treatment. Grade 3–4 adverse events (AEs) were reported in 60% of the patients, almost all of whom were exposed to steroid treatments (59%); AEs were fatal in 4 patients, and led to permanent treatment discontinuation in 31%. Factors significantly associated with outcome were cutaneous histology, low lactate dehydrogenase (LDH), low number of metastatic sites, performance status, first line of treatment and number of combinations administered during the induction phase. *Conclusions:* Despite the profoundly different baseline patient characteristics, the combination of ipilimumab and nivolumab is as effective in the real-world population as it was in clinical trials, including long-term outcomes. In addition to confirming the significance of baseline prognostic factors, our study reveals that the number of combinations effectively administered may also be correlated with good outcome.

## 1. Introduction

The development of immunotherapeutic agents has drastically changed the outlook for melanoma patients over the past decade, significantly increasing survival rates and improving quality of life [1,2,3,4,5]. The anti-Cytotoxic T Lymphocyte Antigen (CTLA)-4 monoclonal antibody, ipilimumab, was the first immune checkpoint inhibitor approved for the treatment of unresectable melanoma [6,7], achieving relatively low response rates but meaningful long-term survival in one fifth of the patients, most of whom were still alive at the 10 year time-point [8]. Shortly after its approval, the Programmed cell death (PD)-1 blocking antibodies pembrolizumab and nivolumab were also approved for the treatment of melanoma [9,10], achieving response rates as high as 26–40%, with superior survival outcomes compared to chemotherapy. PD-1 inhibitors, in turn, showed superiority over ipilimumab in terms of efficacy as well toxicity, having a more favorable safety profile [11]. In addition, PD-1 inhibitors were recently approved as an adjuvant treatment for the American Joint Committee on Cancer (AJCC) stage III melanoma, reducing the hazard ratios for disease recurrence compared to placebo [12] and to ipilimumab by half [13]. The dual blockade of immune checkpoints was evaluated in preclinical studies with ipilimumab and nivolumab, and demonstrated synergic tumor suppression in vivo [14,15]. Successively, early phase clinical trials yielded very promising results [16,17,18] for the combination. CheckMate 067 was the flagship trial of the combinatorial concept [19,20,21], where 945 previously untreated patients with metastatic melanoma were assigned to nivolumab alone, nivolumab and ipilimumab or ipilimumab alone. The combination showed a longer median progression-free survival (PFS) of 11.5 m and a higher rate of response (58%) as compared with either drug alone. A long follow up of 5 years [22] for CheckMate 067 confirmed the durability of the responses (median duration of response for ipilimumab and nivolumab had not been reached at the 5 y landmark) and the consequent plateauing of survival curves, with more than 50% of the patients treated with the combination still alive at that time. Impressively, the median treatment-free interval (time from the last dose of the trial drug to subsequent systemic therapy) was 18.1 months for the ipilimumab-nivolumab group, and the percentage of patients alive and off-treatment without subsequent therapy at 5 y was 74% [22].

Furthermore, impressive results for ipilimumab and nivolumab were reported also for patients with brain metastases, a population with a poor prognoses and a median survival of 4 to 5 months. In CheckMate 204, a phase 2 study [23], asymptomatic patients with at least one nonirradiated brain metastasis of 0.5–3 cm in size achieved an intracranial response rate of 57%, similar to the extracranial response of 56%. Another multicenter Australian study reported a similar intracranial response rate (46%) in asymptomatic patients treated with the combination [24].

These immense achievements, though, are at the cost of toxicity. High grade (grades 3–5) treatment-related AEs are reported in more than half of all patients, with 30% of the cases leading to permanent treatment discontinuation. Attempts to minimize the toxic effect of the combination led to the design of trials with “modified” combination regimens. Specifically, CheckMate 511 evaluated the modified dosing of nivolumab 3 mg/kg + ipilimumab 1 mg/kg and demonstrated a significantly lower incidence of grade 3–5 AEs compared to the standard regimen (35% vs. 48%, *p* = 0.006). Similar safety results were achieved with low dose ipilimumab and pembrolizumab in the keynote 029 phase 1b trial [25]. A recent update with a follow up of 3 years also showed a durable response [26], which was probably similar to the standard-dose regimen; however, this remains to be further evaluated in a randomized fashion.

The aim of our study is to report the efficacy and toxicity outcomes of an out-of-trial, real-life population of melanoma patients treated with the standard dose combination of ipilimumab and nivolumab. The study includes all consecutive patients treated at our institute, including those with poor performance statuses, brain metastases, and those previously treated with other lines of treatment.

## 2. Methods

The study population was comprised of inoperable or metastatic melanoma patients treated with ipilimumab and nivolumab at the Ella Lemelbaum Institute for Immuno-Oncology between January 2014 and May 2019. The data was derived from our melanoma registry—a single center prospectively updated, medical-records based oncologic registry. Eligible cases for analysis had a diagnosis of advanced melanoma and were treated with at least one cycle of the standard dosing combination of ipilimumab 3 mg/kg and nivolumab 1 mg/kg every 3 weeks, followed by maintenance nivolumab 3 mg/kg every 2 weeks. For each patient, the following data were collected: demographics, primary melanoma subtype, disease stage at presentation and BRAF mutation status, as well as baseline characteristics prior to ipilimumab + nivolumab initiation such as disease burden according to AJCC 8th edition staging system, number of metastatic sites, lactate dehydrogenase (LDH) level and Eastern Cooperative Oncology Group Performance Status (ECOG PS). Treatment characteristics included the line of treatment in which ipilimumab + nivolumab was administered as well as previous lines, treatment duration, the number of combinations effectively administered, and reasons for treatment discontinuation. Tumor responses were assessed based on routine radiologic evaluation and clinician determination of response, as reported in the patient’s electronic file. Data on treatment-related adverse events (AE) was collected for all patients, and included the type of AE, onset, grade of severity according to the Common Terminology Criteria for Adverse Events (CTCAE) classification v.5.0, duration, corticosteroid exposure and dosing.

### 2.1. Statistical Analysis

Descriptive statistics were used to describe patient and treatment characteristics. For quantitative variables, we calculated the mean and median with standard deviation and range, respectively. For nominal variables, we calculated frequencies and proportions. Differences among quantitative variables were evaluated using the independent-samples t-test; Pearson’s Chi-square was used to evaluate differences among categorical variables. Associations between prognostic factors and tumor response were assessed with logistic regression. Overall survival (OS) and PFS were estimated from initiation of immunotherapy to death (for OS) and progression or death (for PFS). Patients alive at the last follow-up were censored. For obvious reasons, patients with ocular melanoma were excluded from survival analyses. We used the Kaplan-Meier method to estimate and visualize survival and Cox proportional hazard regressions to assess association with baseline prognostic factors. Significant factors in univariate analysis were considered for multivariate analysis. Statistical significance was defined as *p* ≤ 0.05 level, and all tests were two-sided. All analyses were performed with STATA v.13.0.

### 2.2. Ethics

This single-center, retrospective medical records study was approved by the Institutional Review Board of the Sheba Medical Center (4387-17-SMC).

## 3. Results

### 3.1. Patient Demographics

We identified 172 patients diagnosed with melanoma, who were treated with the combination ipilimumab + nivolumab from January 2014 to July 2019 with a median follow-up of 12 months (range 1–72 m) from treatment initiation. Baseline demographic data are detailed in Table 1. The median age was 59 years, and 99 (58%) patients were male. Most of the patients had cutaneous melanoma, including head-neck and acral melanoma (n-116, 67%), 8 had mucosal melanoma (5%) and 13 (8%) had ocular melanoma. In 35 patients (20%) the disease presented at stage IV without a known primary. Half of the patients (*n* = 85, 49%) had a BRAF V600 mutation, of whom 50 (30%) had a V600E mutation, 3 (2%) had a V600K mutation and 29 (17%) had an unspecified V600 mutation. One hundred and ten (64%) were treatment-naïve patients; of the 62 pretreated patients, 30 (48%) had disease progression on a single agent PD-1 based immunotherapy, and 47 (76%) had failed on targeted therapy with the serine-threonine kinases BRAF and MEK inhibitors. ECOG PS was 0–1 in 149 patients (92%), and 65 (38%) had elevated LDH. The mean number of disease sites was 2.5 (±1.7), and 68 patients (40%) had visceral sites (M1c according to the 8th edition AJCC), of whom 39 had liver involvement. Thirty-eight (22%) had brain metastases at treatment initiation.

Sixty-seven patients (40%) received all four combination treatments, whereas 33 (19%) received only one. The median treatment duration was 18 weeks (range 1–190 w). Reasons for treatment cessation were disease progression in 82 patients (61%), treatment-limiting toxicity in 41 patients (31%), and long-term responses in 7 (5%). At data cutoff, 38 patients (22%) were still on treatment, and 84 (49%) were alive.

### 3.2. Efficacy

The objective response rate (ORR) for the whole population, defined as rate of patients with complete response (CR) and partial response (PR), was 48%. The disease control rate (DCR), defined as rate of patients with CR, PR and stable disease (SD), was 55% (Table 2). Treatment-naïve patients had a higher ORR of 61% with a CR rate of 36%, whereas patients treated in advanced lines had lower ORR and CR rates (25% and 15%, respectively). The median time to best response was 12 weeks (range 0.4–131 weeks). At data cutoff, 101 (64%) patients had disease progression, of whom 31 (19.5%) developed brain metastasis.

**Figure 1:**
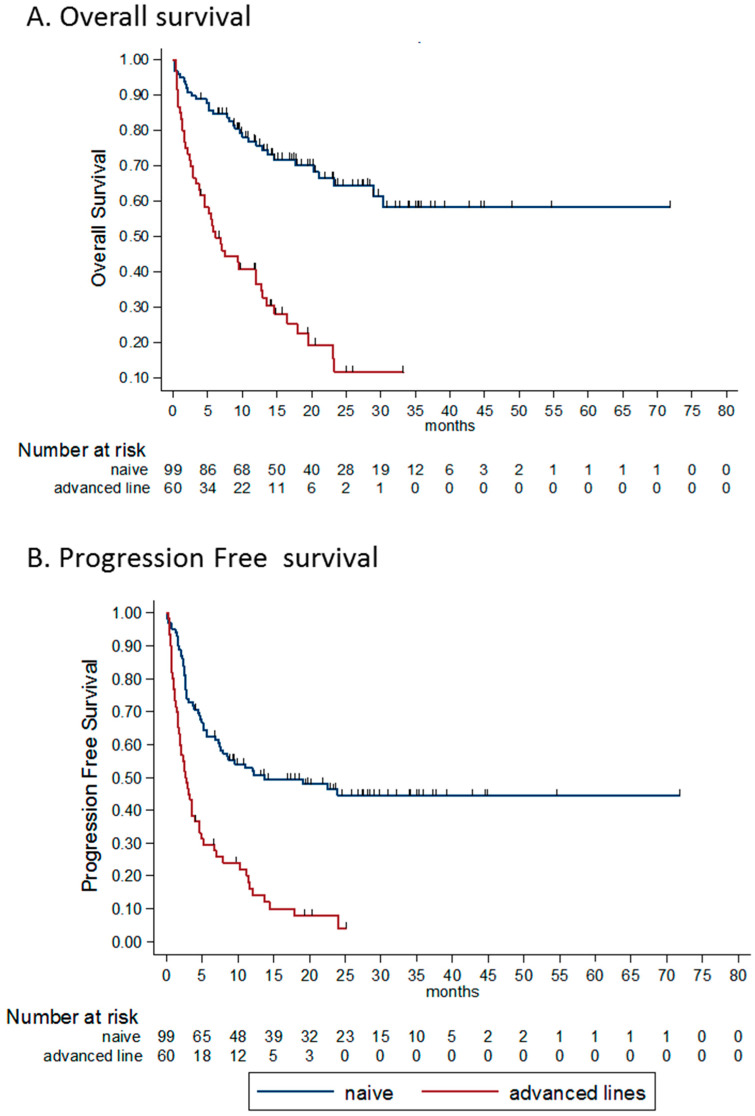

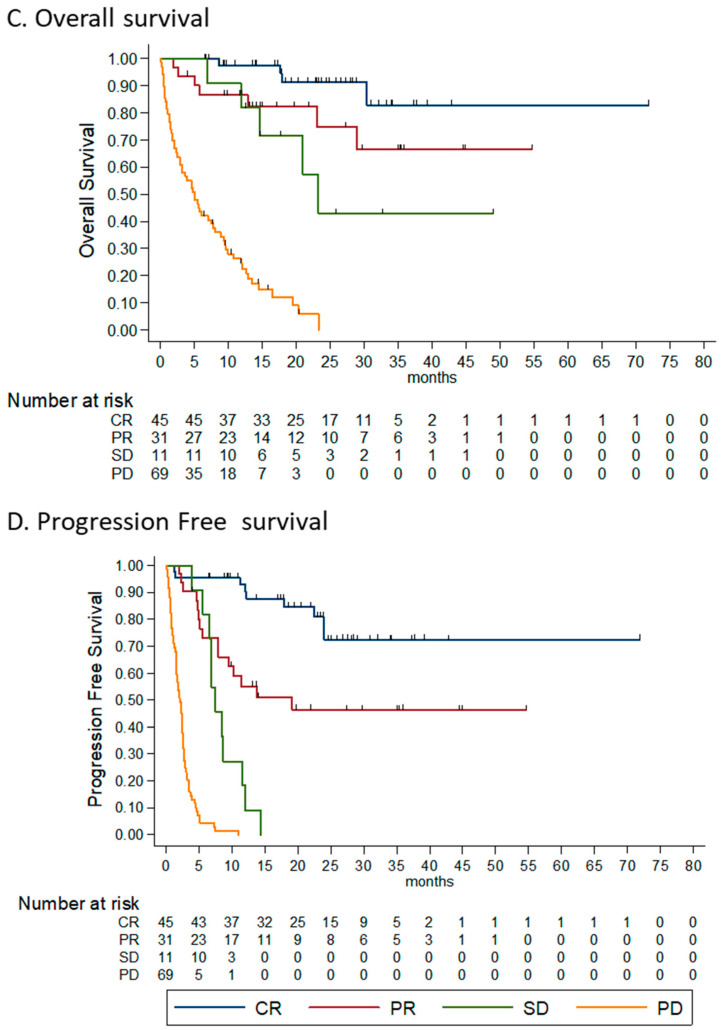
Kaplan-Meier survival estimates according to line of treatment and best response. (**A**) OS according to line of treatment. mOS for naïve patients—NR and 6.1 m for previously treated patients. HR for death 4.0 (95% CI 2.6–6.5, *p* < 0.0001). (**B**) PFS according to line of treatment. mPFS for naïve patients 12.2 m and 2.6 m for previously treated patients. HR for PFS 3.0 (95% CI 2.0–4.5, *p* < 0.0001). (**C**) OS according to best response to the treatment. Mos–NR, NR, 21 m and 4.8 m for CR, PR, SD and PD patients, respectively. The HR for death for patients with PR was 3.06 (95% CI 0.89–10.46 *p* = 0.075) compared CR patients. For patients with SD as best response, HR was 5.79 (95% CI 1.55–21.62, *p* = 0.009) compared to CR patients. (**D**) PFS according to best response to the treatment. mPFS was NR, 19.1 m, 7.5 m and 2.1 m for CR, PR, SD and PD patients, respectively. HR for PFS for patients with PR was 3.45 (95% CI 1.5–7.9, *p* = 0.035) compared to CR patients. For patients with SD as best response, HR for PFS was 11.62 (95% CI 4.57–29.55, *p* < 0.0001) compared to CR patients. OS—overall survival, PFS—progression free survival, HR—hazard ratio, NR not reached, CR complete response, PR—partial response, SD—stable disease, PD—progressive disease.

The median PFS for treatment-naïve patients was 12.2 months, and 2.6 months for patients treated in advanced lines. The estimated rates of PFS for treatment-naïve patients was 52% (95% CI 41–61) at one year and 45% (95% CI 34–55) at both two and three years. The estimated PFS rates for previously treated patients at one and two years were 14% (95% CI 6–25) and 4% (95% CI 0.5–14), respectively. The hazard ratio (HR) for progression or death was 3.0 (95% CI 2.0–4.5, *p* < 0.0001; Figure 1A,B).

The median PFS for patients who had CR as best response was not reached (NR), whereas for patients with PR, SD and progressive disease (PD), the median PFS were 19.1 m, 7.5 m and 2.1 m, respectively. The HR for progression or death for patients with PR was 3.45 (95% CI 1.5–7.9, *p* = 0.035) compared to patients with CR. For patients with SD as best response, HR for progression or death was significantly higher compared to patients that achieved CR with 11.62 (95% CI 4.57–29.55, *p* < 0.0001; Figure 1C,D).

The median OS for treatment-naïve patients was NR, and 6.1 months for patients treated in advanced lines. The estimated rates of OS for treatment-naïve patients at one, two and three years were 76% (95% CI 66–83), 64% (95% CI 53–74) and 58% (95% CI 45–70), respectively. The estimated OS rates for previously treated patients at one, two and three years were 37% (95% CI 24–49%), 12% (95% CI 4–25) and 12% (95% CI 4–25), respectively. The HR for death was 4.0 (95% CI 2.6–6.5, *p* < 0.0001).

The median OS for patients who had CR as best response was NR, whereas for patients with PR, SD and PD median OS was NR, 21 m and 4.8 m, respectively. The HR for death for patients with PR was 3.06 (95% CI 0.89–10.46 *p* = 0.075) compared to patients with CR. For patients with SD as best response, HR was higher compared to patients that achieved CR 5.79 (95% CI 1.55–21.62, *p* = 0.009).

### 3.3. Toxicity

**Figure 2:**
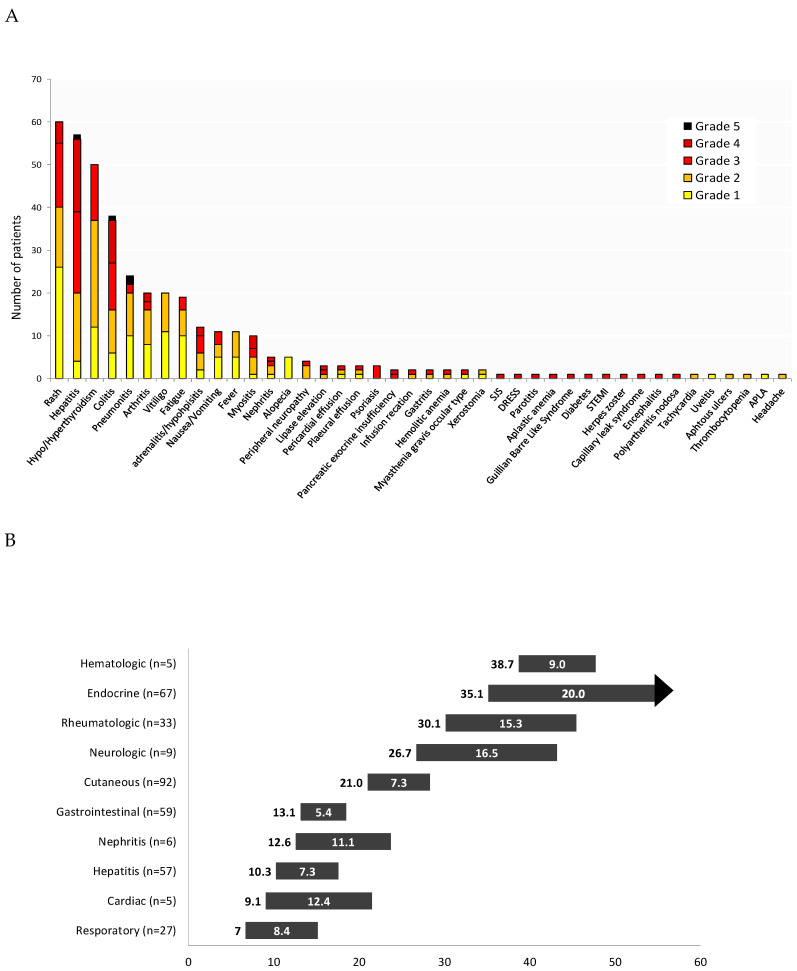
(**A**). Treatment-related adverse events in patients treated with ipilimumab and nivolumab. (**B**). Median onset and median duration of immune related adverse events (weeks).

AEs of any grade were reported in 90% of the patients (Figure 2A). The most frequent AEs were rash (*n* = 60, 35%), followed by hepatitis (*n* = 57, 33%), thyroid dysfunction (*n* = 50, 29%) and colitis (*n* = 38, 22%). Grade 3–4 toxicity was reported in 103 patients (60%). The most frequent grade 3–4 AEs were hepatitis (22%), followed by colitis (13%) and rash (12%). Four patients (2%) died as a result of a complicated AE: two patients with pneumonitis, one with hepatitis, and one with colitis. The shortest median time to occurrence of AEs (Figure 2B) was seven weeks for respiratory AEs (27 cases), followed by cardiac AEs (5 cases, 9.1 weeks) and hepatitis (57 cases, 10.3 weeks). The longest median time to occurrence of AEs was 38.7 weeks for hematologic AEs (5 cases) followed by endocrine AEs (67 cases, 35.1 weeks) and rheumatologic AEs (33 cases, 30.1 weeks). The shortest median duration of AEs was for gastrointestinal AEs (59 cases, 5.4 weeks), whereas the longest was for neurological AEs (9 cases, 16.5 weeks). Endocrine AEs were mostly permanent, as expected. Adverse events led to permanent treatment discontinuation in 41 patients (31%).

The majority of the patients (*n* = 102, 59%) were treated with steroid therapy, of whom 32 (31%) received intravenous methylprednisolone (Table 3). The mean prednisolone-equivalent dose was 1.7 mg/kg (±2.3), and the median duration of steroid treatment was 12 weeks (range 1–153 weeks). Eleven patients (6%) were treated with advanced immune-suppression (infliximab, mycophenolate mofetil, methotrexate, cyclosporine, intravenous immunoglobulin and plasma exchange).

### 3.4. Factors Associated with Outcome

We examined the differences in baseline characteristics between patients who achieved an objective response (*n* = 76) and those who did not achieve objective response (non-responders, defined as patients who achieved SD or PD as best response, *n* = 80). We also examined the association of these factors with survival outcomes.

#### 3.4.1. Histology Subtype

As expected, melanoma subtype was found to be significantly related to the probability of response. Specifically, patients with mucosal melanoma had a lower response rate compared to those with cutaneous melanoma (12% vs. 50%, respectively; *p* = 0.04). Furthermore, the median OS for cutaneous melanoma was 28.9 m, whereas it was 6.3 m for mucosal melanoma (HR for death 3.13, 95% CI 1.41–6.92, *p* = 0.005).

#### 3.4.2. Disease Burden

Elevated LDH rate was higher in the non-responders groups with a mean LDH ratio of 2.12 ± 2.71 vs. upper normal limit (UNL) compared to 1.0 ± 0.6 in the responders group (*p* = 0.003). High LDH was associated with a lower probability of response (OR 0.43, 95% CI 0.25–0.76, *p* = 0.003), with HR for death of 1.2 (95% CI 1.13–1.28, *p* < 0.0001). The number of metastatic sites was also significantly different among groups, where responders had a mean value of 2.13 ± 1.33 sites and non-responders 2.86 ± 1.93 sites (*p* = 0.005). A higher number of metastatic sites was also associated with poorer survival (HR 1.26, 95% CI 1.13–1.41, *p* < 0.0001).

#### 3.4.3. ECOG Performance Status

Performance status scores were significantly different between responders and non-responders. ECOG PS scores of 1 and ≥2 were associated with a significantly lower probability of response, as compared to patients with ECOG PS = 0 (OR 0.33, 95% CI 0.14–0.75, *p* = 0.008 and OR 0.07, 95% CI 0.01–0.63, *p* = 0.017, respectively). Median OS for patients with ECOG PS = 0 was NR, whereas for patients with PS = 1 and ≥2, the median OS rates were 5.9 m and 2.1 m, respectively (*p* < 0.0001).

#### 3.4.4. Line of Treatment

Patients receiving ipilimumab and nivolumab as an advanced line of treatment had a significantly lower probability of response compared to the first-line setting (OR 0.21, 95% CI 0.10–0.43, *p* < 0.0001). The overall and progression-free survival outcomes (Figure 1) were also significantly affected by the treatment line (HR 4.0 (95% CI 2.6–6.5), *p* < 0.0001) and HR 3.0 (95% CI 2.0–4.5), *p* < 0.0001, respectively).

#### 3.4.5. Number of Combinations Administered

**Figure 3:**
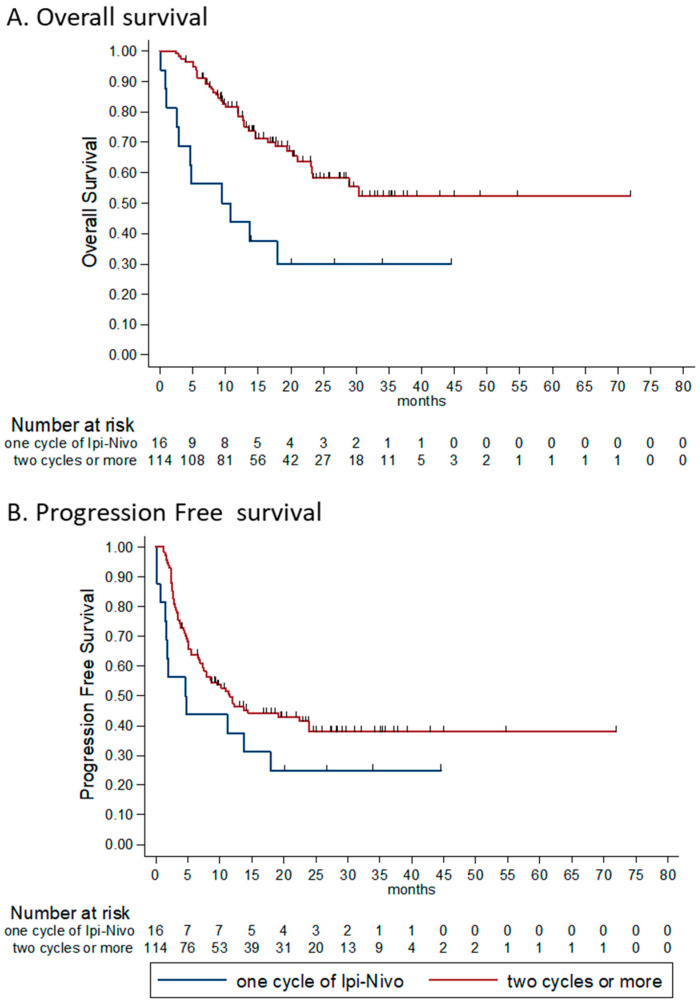
Kaplan-Meier survival estimates according to number of combinations received in the induction phase (stopped for toxicity only). (**A**) OS according to number of ipilimumab and nivolumab combinations received. HR for death 0.35 (95% CI 0.18–0.68, *p* = 0.002) for patients receiving two or more cycles, compared to patients who received only one cycle. Median OS were NR and 9.5 m, respectively. (**B**) PFS according to number of ipilimumab and nivolumab combinations received. HR for progression or death 0.58 (95% CI 0.31–1.07, *p* = 0.085) for patients receiving two or more cycles, compared to patients who received only one cycle. OS—overall survival, PFS—progression free survival, HR—hazard ratio.

Within the patients whose disease did not progress during the induction phase (*n* = 130), we found that the number of combinations ipilimumab-nivolumab received had a predictive effect on survival. The reason for discontinuation (permanently or switching to monotherapy) was toxicity. We found that patients who received two or more cycles had statistically significant longer OS compared to patients who received only one cycle; median OS were NR and 9.5 m, respectively (HR 0.35, 95% CI 0.18–0.68, *p* = 0.002). Furthermore, the PFS for patients who received two or more cycles was borderline statistically significantly longer compared to those who received only one cycle (HR for PFS 0.58, 95% CI 0.31–1.07, *p* = 0.085; Figure 3).

#### 3.4.6. BRAF Status

Considering patients with a known BRAF status who received ipilimumab + nivolumab in the first-line setting (95 patients, 60%), more than a third (*n* = 37, 39%) had a BRAF V600 mutation and 58 (61%) had BRAF wild type (WT).

Response rate for ipilimumab and nivolumab given the first line setting was significantly higher for BRAF mutant patients compared to BRAF WT (70% vs. 57%, *p* = 0.015). Accordingly, there was a borderline significant favorable survival outcome for BRAF mutant patients treated with ipilimumab and nivolumab in the first line setting, compared to BRAF WT; HR for OS was 0.5 (95% CI 0.22–1.13, *p* = 0.096) and HR for PFS was 0.53 (95% CI 0.27–1.03, *p* = 0.061).

#### 3.4.7. Toxicity and Steroid Treatment

In order to analyze the effect of toxicity and immunosuppression on efficacy functions, we omitted from the analysis patients for whom no AE was reported (17 patients, 10% of the population), because their median survival time was 1.3 m (interquartile range 0.5–3.2 m), and so they did not have the time to develop any AEs (bias).

**Figure 4:**
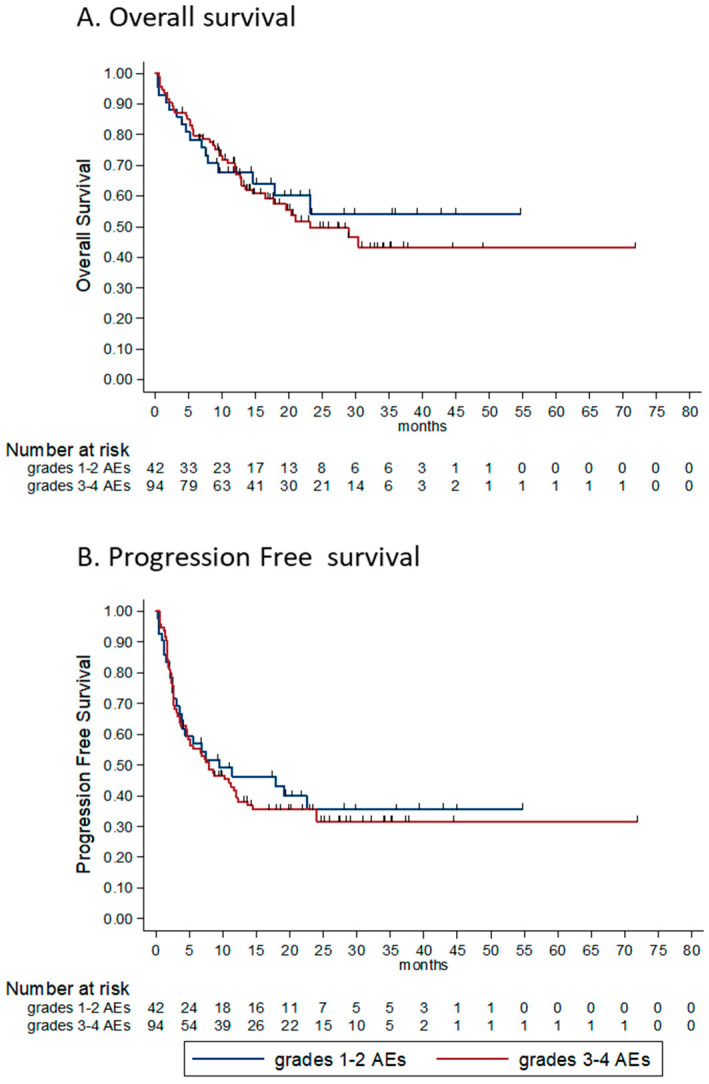

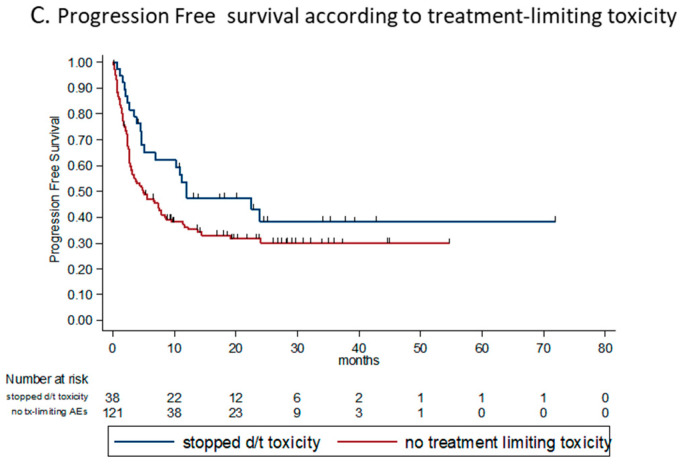
Kaplan-Meier survival estimates according to maximal grade of adverse event and according to treatment discontinuation. (**A**) OS according to maximum grade of AE. HR for death was 1.09 (95% CI 0.62–1.95, *p* = 0.746) for patients experiencing grade 3–4 AEs compared to those who experienced grade 1–2 AEs. (**B**) PFS according to maximal grade of AE. HR for progression or death was 1.08 (95% CI 0.68–1.72, *p* = 0.739) for patients experiencing grade 3–4 AEs compared to those who experienced grade 1–2 AEs. (**C**) PFS according treatment limiting toxicity (TLT)—patients who had to discontinue therapy due to TLT, had a numerically longer PFS compared to patients who did not experience TLT (median PFS 12 m vs 4.9 m, respectively; HR for progression or death for patients without TLT was 1.55 (95% CI 0.96–2.52), *p* = 0.07. OS—overall survival, AE—adverse event, HR—hazard ratio, PFS—progression free survival.

We hypothesized that the maximal severity of AE experienced would be associated with outcome, yet found that it was associated with neither response nor overall survival or progression-free survival. The HR for OS and for PFS were 1.09 (95% CI 0.62–1.95, *p* = 0.746) and 1.08 (95% CI 0.68–1.72, *p* = 0.739) for patients experiencing grade 3-4 AEs compared to those who experienced grade 1–2 AEs, respectively (Figure 4A,B).

The rate of patients exposed to steroids due to AEs was not different between responders and non-responders (64% vs. 54%, respectively, *p* = 0.173), and exposure to steroids did not seem to have an effect of overall survival (HR for OS 0.67 (95% CI 0.40–1.10), *p* = 0.116) nor on PFS (HR 0.91 (95% CI 0.58–1.40), *p* = 0.661). Looking at the duration of steroid treatment and maximal dosage, surprisingly, we found that the median duration was significantly longer among the responders (15 weeks vs 11 weeks, *p* = 0.04), and the maximal dose of steroids was numerically higher in the non-responders (2.1 mg/kg vs. 1.3 mg/kg, *p* = 0.06).

Though only borderline statistically significant, we noticed that patients who had to discontinue therapy due to treatment-limiting toxicity (TLT) had a longer PFS compared to those who did not experience TLT (median PFS 12 m vs. 4.9 m, respectively; HR for progression or death for patients without TLT was 1.55, 95% CI 0.96–2.52, *p* = 0.07; descriptive data in Figure 4C).

#### 3.4.8. Uni- and Multivariable Analysis

In our multivariable cox regression analysis for overall survival, we included variables that were significant in the univariable analysis. For a full list of variables and their respective HR for OS, see Table 4. Factors that were significantly associated with survival in the multivariable analysis were ECOG PS (*p* < 0.0001), line of treatment (*p* = 0.003), the number of combinations administered in the induction phase (*p* = 0.005) and best tumor response (*p* < 0.0001).

## 4. Discussion

The results of this trial shed light on the real-life outcomes of treatment with a combination of ipilimumab and nivolumab in metastatic melanoma patients, as seen in the routine daily clinic. This population is profoundly different from the typical population enrolled in clinical studies, given the strict inclusion-exclusion criteria. Our study population also included patients with mucosal and ocular melanomas (13%), with brain metastasis (22%) and patients receiving the treatment in a second line or higher (36%). Despite having elevated LDH levels (38%), most of the population was generally in good PS (86% had ECOG 0–1).

The results for the cohort treated in the first line setting (*n* = 99, 62%) seemed to be comparable to those reported in the pivotal trial Checkmated 067 [19], with a response rate of 61% and CR rate of 36%. Responses were prolonged, with median OS and PFS for this population being not-reached and 12.2 m, respectively. The long follow-up shows the typical plateau of the survival curves starting at the two–three year time-point, similar to the long-term results of the prospective clinical trials [18,20,21,22].

Patients harboring BRAF V600 mutations treated with ipilimumab and nivolumab in the first line seemed to achieve a higher response rate compared to the BRAF-WT population. There was also a trend towards more favorable PFS and OS outcomes. This may be a result of the oncologists in our institution assigning the “good” BRAF-mutant patients to immunotherapy rather than to targeted therapy, saving the last option for disease progression. Another explanation would simply be the superiority of this regimen in BRAF mutant patients, as was also shown in the CheckMate 067 trial, where median OS for patients with and without BRAF mutations was not-reached and 39.1 months, and median PFS were 16.8 and 11.2 months, respectively. Similar results were recently reported for BRAF mutant patients in a real-world study [27]. On the other hand, other phase 3 trials demonstrated similar outcomes for BRAF mutant and WT with immunotherapy. To date, no formal head-to-head trial has been published, and the question of the best first line choice for BRAF mutant patients hasn’t been answered. Hopefully, the results of the ongoing phase 3 trials DREAMseq and SECOMBIT will solve this dilemma [28,29].

More than a third of the study population (62 patients, 38%) had disease progression on prior treatments before receiving ipilimumab and nivolumab. The outcome in this population was significantly poorer compared to the first-line population, i.e., lower response rates (25%) and shorter PFS (median 2.6 m) and OS (median 6.1 m). These observations confirm the importance of treatment selection in the first line setting. The response rate to ipilimumab and nivolumab administered in advanced lines for patients previously exposed to PD-1 inhibitors (30 patients) or to BRAF-MEK inhibitors (47 patients) were 23% and 23%, respectively. Retrospective series have shown similar results for ipilimumab and nivolumab given after progression on anti-PD-1, including the most recent Australian work presented at the ASCO 2020 [30], where 355 patient whose disease progressed on anti PD-1 were treated with either ipilimumab and anti-PD1 or with ipilimumab alone. The RR for the combination was significantly higher (32% vs. 13%, respectively, *p* = 0.0021). The final results from a prospective phase 2 trial were also presented at the same ASCO meeting by Olson [31], where 70 patients resistant to anti PD-1 (10 in the adjuvant setting) received pembrolizumab + low dose ipilimumab and achieved a RR of 30%. The administration of ipilimumab and nivolumab in the second line after progression on BRAF and MEK inhibitors is described by Mason et al., reporting a response rate of 21% and median PFS of merely two months [32].

Our study confirms the influence of pathological, radiological and clinical factors on survival and tumor responses [33,34]. Specifically, we show that the histologic type of melanoma, the disease burden (expressed as the number of disease sites and by LDH) and ECOG PS prior to treatment initiation were all significantly associated with tumor response, PFS and OS. Furthermore, the best tumor response achieved was significantly associated with survival outcomes; complete responders (28% of our study population) achieved impressively good results compared to non-CR patients, having 3y OS and 3y PFS of 83% and 72%, respectively. The long-term results for 82 complete responders treated with ipilimumab and nivolumab in three key trials were presented at the 2017 ESMO meeting, showing 3y OS and PFS of 90% and 80%, respectively [35]. These results are naturally superior to those achieved by our population, representing the differences between study and real-world baseline patient characteristics.

The number of combination cycles during the induction phase should be four per-protocol, while, effectively, only 40% received all four, and as many as 45% received only one or two. The median number of combination cycles effectively administered in randomized clinical trials was reported to be four [18,19]. Real-world studies, in contrast, report a lower number of combinations, in line with our findings [36,37]. Interestingly, among those who did not have disease progression during the induction phase (*n* = 130), we found that the number of combinations administered was associated with survival, and so according to our data, patients receiving only one cycle of ipilimumab-nivolumab and then continuing to receive maintenance monotherapy may have inferior outcomes compared to patients receiving more than two cycles of ipilimumab-nivolumab.

On the other hand, we noticed that patients who had to permanently discontinue therapy due to AEs at any point during the course of treatments (treatment limiting AEs, TLT) seemed to have a borderline-significantly longer PFS compared to those who did not experience TLT. These results are in line with reports from pivotal randomized trials [18,38] and with other real-life data for ipilimumab and nivolumab [36]. A pooled analysis from randomized phase 2 and 3 trials by Schadendorf et al. reports favorable outcomes for patients who permanently discontinued treatments in the induction phase due to toxicity compared to patients who did not [39]. These results, put together, allow us to conclude that many patients may continue to derive benefit from the treatment even after discontinuation, but that the number of combinations given in the induction phase may also play a role in the long-term outcome.

High-grade treatment-related toxicity was reported in 60% of the population, as expected from this regimen [40,41,42,43]. Nearly a third of the patients (31%) had to permanently discontinue treatments due to AEs, and mortality due to AEs was 2.3%. These numbers undoubtedly affect both patients and the health system. These results are in line with the report of Joseph et al., showing that patients treated with ipilimumab and nivolumab were more likely to be hospitalized, had more than one hospitalizations due to AEs and had longer hospitalization times compared to patients treated with monotherapy [44]. We analyzed the grade of severity of AEs in the context of efficacy. After excluding patients for whom no AE were reported due to short survival times, we found no association between grade of AE and tumor response, nor with survival. In fact, patients experiencing mild–moderate (grades 1–2) AEs had similar outcomes as those experiencing severe AEs (grades 3–4). Furthermore, in contrast to the common belief and to other retrospective real-life data on ipilimumab and nivolumab [45], exposure to steroids (59% of our cohort) due to AEs was not associated with poorer outcome and did not seem to compromise the long-term immune response. Interestingly though, the maximal dose of steroids was numerically higher within the non-responders, although this was not statistically significant. High dose steroidal therapy, especially in the induction phase, should therefore be further investigated.

## 5. Conclusions

The combination of ipilimumab and nivolumab is as effective in the real-world population as it has been in clinical trials, including regarding long-term outcomes. Toxicity is not higher than in clinical trials, and is manageable. Factors associated with efficacy were the line of treatment, best tumor response, low disease burden and good PS. The number of combinations received in the induction phase may also affect the outcome.

## Figures and Tables

**Table 1:** Baseline patient characteristics.

Characteristics	All Patients N = 172	Non Responders (SD/PD), *n* = 90	Responders (CR/PR), *n* = 79	*p*-Value
Age (median, range)	59 (12–80)	59 (12–80)	60 (12–80)	0.635
Male (%)	99 (58%)	52 (58%)	46 (58%)	0.953
Melanoma subtype (%)				
Cutaneous	116 (67%)	58 (50%)	58 (50%)	-
Mucosal	8 (5%)	7 (88%)	1 (12%)	0.04 *
ocular	13 (8%)	10 (77%)	3 (23%)	0.065 *
Unknown primary	35 (20%)	15 (43%)	17 (49%)	0.754 *
Disease presentation				
Advanced upfront Recurrent disease	46 (28%) 124 (72%)	21 (46%) 69 (56%)	22 (48%) 55 (44%)	0.235
BRAF wild-type	81 (47%)	43 (53%)	38 (47%)	-
BRAF V600 mutant	85 (49%)	44 (52%)	38 (45%)	0.984
V600E mutation	50 (30%)	26 (52%)	24 (48%)	
V600K mutation	3 (2%)	1 (33%)	2 (67%)	
V600 unspecified	29 (17%)	17 (59%)	12 (41%)	
Unknown status	6 (3%)	3 (50%)	3 (50%)	
LDH				
Ratio (mean±SD) > ULN (%)	1.59 ± 2.08 65 (38%)	2.12 ± 2.71 41 (63%)	1.0 ± 0.6 23 (35%)	0.003
AJCC 8th edition (%)				
IIIC	2 (1%)	0	2 (3%)	-
M1a	32 (19%)	16 (50%)	16 (50%)	
M1b	32 (19%)	16 (50%)	16 (50%)	1.000
M1c	68 (39%)	37 (54%)	29 (43%)	0.573
M1d	38 (22%)	21 (55%)	16 (42%)	0.575
Number of disease sites (mean ± SD)	2.5 ± 1.7	2.86 ± 1.93	2.13 ± 1.33	0.005
ECOG PS (%)				
0	114 (66%)	46 (40%)	66 (58%)	-
1	35 (20%)	23 (66%)	11 (31%)	0.008 **
≥2	13 (8%)	12 (92%)	1 (8%)	0.017 **
unknown	10 (6%)	9 (90%)	1 (10%)	
Treatment naïve	110 (64%)	43 (39%)	64 (58%)	
Previously treated	62 (36%)	47 (76%)	14 (23%)	<0.001
Previous treatments				-
Immunotherapy	30 (17%)	23 (77%)	7 (23%)	
BRAF inhibitors	47 (27%)	36 (77%)	11 (23%)	
N° of comb Ipi-Nivo (%)				
All four	67 (40%)	27 (40%)	40 (60%)	
2–3	72 (42%)	38 (53%)	34 (47%)	-
Only one	33 (19%)	25 (76%)	5 (15%)	-
Weeks on treatment (median, range)	18 (1–190)	9 (1–57)	49 (1–190)	-

CR—complete response, PR—partial response, SD-stable disease, PD—progressive disease, LDH—Lactate Dehydrogenase; AJCC—American Joint Committee on Cancer; ECOG PS—Eastern Cooperative Oncology Group Performance Status; Ipi-Nivo—Ipilimumab and Nivolumab combination. * compared to cutaneous melanoma, ** compared to ECOG PS score = 0.

**Table 2:** Response rates to combined ipilimumab and nivolumab *.

Response	All Patients (*n* = 159)	Treatment Naïve (*n* = 99)	Advanced Lines (*n* = 60)
CR	45 (28%)	36 (36%)	9 (15%)
PR	31 (20%)	25 (25%)	6 (10%)
SD	11 (7%)	6 (6%)	5 (8%)
PD	69 (43%)	29 (29%)	40 (67%)
NE	3 (2%)	3 (3%)	0
DCR	55%	67%	33%
**ORR**	**48%**	**61%**	**25%**

CR—complete response, PR—partial response, SD—stable disease, PD—progressive disease, NE—non evaluable, DCR—disease control rate; ORR—overall response rate. * excluded ocular melanoma patients (*n* = 13).

**Table 3:** Toxicity and immunosuppression.

Characteristics	All Patients, N = 172	Non Responders (SD/PD), *n* = 90	Responders (CR/PR), *n* = 79	*p*-Value
Maximal severity of AE *, (%)				
None	17 (10%)	15 (17%)	1 (1%)	-
Grade 1–2	45 (28%)	22 (24%)	23 (29%)	-
Grade 3–4	103 (60%)	52 (58%)	50 (66%)	0.901 ^¥^
Grade 5	4 (2%)	1(1%)	3 (4%)	^-^
Steroid treatment, (%)	102 (59%)	51 (57%)	50 (63%)	0.381
Duration of steroid treatment, weeks—median (range)	12 (1–153)	12 (1–106)	16 (1–153)	0.039
Maximal dose of steroids, mg/kg **—mean ± SD	1.7 ± 2.3	2.1 ± 3.0	1.3 ± 1.2	0.060
Advanced immune suppression ^†^ (%)	11 (6%)	5 (6%)	6 (8%)	0.592

AE adverse event. * According to the Common Terminology Criteria for Adverse Events (CTCAE) classification v5.0. ** Oral prednisolone equivalent dose. ^†^ Infliximab, mycophanolate mofetil, methotrexate, cyclosporine, intravenous immunoglobulin (IVIG) or plasma exchange. ^¥^ grades 3–4 vs. 1–2.

**Table 4:** Uni-and multi- variable analysis.

Variable	Univariable Analysis		Multivariable Analysis	
	HR for Death (95% CI)	*p*-Value	HR for Death (95% CI)	*p*-Value
Histology subtype (mucosal vs. cutaneous)	3.13 (1.41–6.92)	0.005	1.84 (0.69–5.04)	0.217
LDH ratio	1.20 (1.13–1.28)	0.003	1.07 (0.97–1.18)	0.176
Number of metastatic sites	1.26 (1.13–1.41)	<0.0001	1.14 (0.98–1.34)	0.092
ECOG PS	3.02 (2.27–4.01)	<0.0001	2.00 (1.37–2.90)	<0.0001
Line of treatment	4.06 (2.56–6.46)	<0.0001	2.70 (1.41–5.15)	0.003
Number of combinations administered	0.62 (0.51–7.66)	<0.0001	0.68 (0.52–0.89)	0.005
BRAF status (V600 mutant vs. WT)	0.5 (0.22–1.13)	0.096		
Best tumor response (NR vs. R)	12.42 (6.43–24.00)	<0.0001	8.67 (3.69–20.39)	<0.0001
Grade of AEs (3–4 vs. 1–2)	1.09 (0.62–1.95)	0.746		
Exposure to steroids	0.67 (0.40–1.10)	0.116		

HR—hazard ratio, CI—confidence interval, LDH—lactate dehydrogenase, ECOG PS—Eastern Cooperative Oncology Group Performance Status, WT—wild type, NR—nonresponders (stable- and progressive-disease), R—responders (complete- and partial-response), AE—adverse events.
